# PTTG3P promotes gastric tumour cell proliferation and invasion and is an indicator of poor prognosis

**DOI:** 10.1111/jcmm.13239

**Published:** 2017-06-19

**Authors:** Weiwei Weng, Shujuan Ni, Yiqin Wang, Midie Xu, Qiongyan Zhang, Yusi Yang, Yong Wu, Qinghua Xu, Peng Qi, Cong Tan, Dan Huang, Ping Wei, Zhaohui Huang, Yuqing Ma, Wei Zhang, Weiqi Sheng, Xiang Du

**Affiliations:** ^1^ Department of Pathology Fudan University Shanghai Cancer Center Shanghai China; ^2^ Department of Oncology Shanghai Medical College Fudan University Shanghai China; ^3^ Institute of Pathology Fudan University Shanghai China; ^4^ Department of Pathology Obstetrics and Gynecology Hospital of Fudan University Shanghai China; ^5^ Wuxi Oncology Institute the Affiliated Hospital of Jiangnan University Wuxi Jiangsu China; ^6^ Department of Pathology First Hospital Affiliated to Xinjiang Medical University Urumqi China; ^7^ Institutes of Biomedical Sciences Fudan University Shanghai China

**Keywords:** PTTG3P, gastric cancer, prognosis, proliferation, invasion

## Abstract

Pseudogenes play a crucial role in cancer progression. However, the role of pituitary tumour‐transforming 3, pseudogene (PTTG3P) in gastric cancer (GC) remains unknown. Here, we showed that PTTG3P expression was abnormally up‐regulated in GC tissues compared with that in normal tissues both in our 198 cases of clinical samples and the cohort from *The Cancer Genome Atlas* (TCGA) database. High PTTG3P expression was correlated with increased tumour size and enhanced tumour invasiveness and served as an independent negative prognostic predictor. Moreover, up‐regulation of PTTG3P in GC cells stimulated cell proliferation, migration and invasion both *in vitro* in cell experiments and *in vivo* in nude mouse models, and the pseudogene functioned independently of its parent genes. Overall, these results reveal that PTTG3P is a novel prognostic biomarker with independent oncogenic functions in GC.

## Introduction

Gastric cancer (GC) is one of the most common malignancies in the world, ranking as the second most prevalent carcinoma and the third leading cause of cancer‐related death in China 1. As it is difficult to detect at early stage, GC often leads to unresectable primary tumours and poor chemotherapy effects. Therefore, a better understanding of the general genetic profile associated with the pathogenesis and progression of GC is urgently needed.

Pseudogenes, genomic *loci* that resemble real genes, were once regarded as functionless entities, harbouring premature stop codons, deletions/insertions or frameshift mutations that abrogate the normal transcription and translation of ‘real’ genes 2. In recent years, however, several studies have shown that pseudogenes also play critical roles in tumourigenesis/tumour suppression by competing with the expression of their true gene counterparts or through processing parent gene‐targeted siRNAs 2, 3. Subsequently, various pseudogenes that are critically involved in carcinogenesis and cancer progression have been disclosed 4, 5, 6, 7, but investigation into their functions in GC remains limited.

Pituitary tumour‐transforming 3, pseudogene (PTTG3P), an intronless gene that is highly homologous to its family members pituitary tumour‐transforming 1 (PTTG1) and pituitary tumour‐transforming 2 (PTTG2), was first identified by Kakar and colleagues in 2000 8. Both PTTG1 and PTTG2 have been reported to serve oncogenic functions in human cancers 9, 10, 11, but the role of PTTG3P in GC remains unclear, and this pseudogene has previously been regarded as functionless.

In this study, we assessed PTTG3P expression using our previously described microarray analysis 12 and subsequently validated its expression in GC tissue specimens. We found that PTTG3P was significantly up‐regulated in GC tissues and served as an independent risk factor for poor disease‐free survival (DFS) and overall survival (OS). In addition, PTTG3P overexpression stimulated cell proliferation, potentially by inducing the G_1_–S transition, and promoted cell invasion both *in vitro* and *in vivo*. Moreover, the expression and function of PTTG3P were found to be independent of its true gene counterpart. Overall, these results reveal that PTTG3P is a novel prognostic predictor in GC that might enable the development of new therapeutic strategies for GC.

## Materials and methods

### Patients

A retrospective cohort study was conducted among 198 patients who underwent surgical resection of primary gastric carcinoma from 2008 to 2010 at the Fudan University Shanghai Cancer Center. GC diagnoses were histopathologically confirmed. None of the patients received pre‐operative therapy. The resected tissue samples were immediately frozen in liquid nitrogen and stored at −80°C until RNA extraction. Clinicopathological features from all patients were obtained from medical records, pathological reports and personal interviews; these included age, gender, DFS, OS and tumour features (such as tumour location, size, differentiation, depth of invasion and the presence/absence of lymphatic metastasis). GC stage was defined according to the TNM classification system. The patients were followed up every 3 months during the first year after surgery and every 3–6 months thereafter until 31 May 2015. All patients had complete follow‐up information. DFS was calculated from the date of surgery to the date of disease progression (local and/or distal tumour recurrence) or to the date of death. OS was defined as the length of time between surgery and death of the patient or the last follow‐up date. The study was approved by the Clinical Research Ethics Committee of the Fudan University Shanghai Cancer Center. Written informed consent was obtained from all participants for the use of their tissues in this study.

### RNA isolation, reverse transcription and quantitative real‐time PCR

Total RNA was extracted from tissue samples and cell lines using TRIzol reagent (Invitrogen, Carlsbad, CA, USA) according to the manufacturer's protocol. Reverse transcription (RT) and quantitative real‐time PCR (qRT–PCR) kits (Takara, Dalian, China) were utilized to evaluate PTTG3P expression. RT and qRT–PCR were performed as previously described 13. Specific primer sets for PTTG1, PTTG2 and PTTG3P were designed based on regions with low homology among these genes. PCR of the relevant genes and sequencing of the PCR products were carried out to ensure specificity. β‐actin was included as an endogenous control to normalize the data.

The primer sequences used in this study were as follows: 5′‐TGACTGTTCCGCTGTTTAGC‐3′ (forward) and 5′‐TAAGGCTTTGATTGAAGGTCCAG‐3′ (reverse) for PTTG1; 5′‐ATTGGAGAACCAGGCACC‐3′ (forward) and 5′‐CGTCGTGTTAAAACTTGAGATA‐3′ (reverse) for PTTG2; 5′‐AAACGAAGAACCAGGCATCCTT‐3′ (forward) and 5′‐GGGAGCATCGAATGTTTTGCC‐3′ (reverse) for PTTG3P; and 5′‐TCCTCTCCCAAGTCCACACA‐3′ (forward) and 5′‐GCACGAAGGCTCATCATTCA‐3′ (reverse) for β‐actin.

### Cell lines and culture conditions

HEK‐293T and HEK‐293FT cells were cultured in Dulbecco's modified Eagle's medium (Gibco, Carlsbad, CA, USA) supplemented with 10% foetal bovine serum (FBS) (Gibco), 50 U/ml penicillin and 50 μg/ml streptomycin (Gibco). The human GC cell lines AGS, HGC‐27, MGC‐803, MKN‐45 and SGC‐7901 were cultured in RMPI‐1640 (Gibco) supplemented with 10% foetal bovine serum (FBS) (Gibco), 50 U/ml penicillin and 50 μg/ml streptomycin (Gibco). All cell lines were maintained at 37°C and 5% CO_2_ in a humidified atmosphere.

### Transient transfection and lentivirus transduction

The full‐length PTTG3P sequence was amplified by PCR from the cDNA of HEK‐293T cells and then subcloned into a pcDNA3.1 (+) vector (Transheep, Shanghai, China). pHBLV‐IRES‐ZsGreen‐PGK‐puro lentivirus was constructed by Hansheng (Shanghai, China).

Viral particles were harvested 48 hrs after cotransfection of the pHBLV‐IRES‐ZsGreen‐PGK‐puro constructs, the packaging plasmid ps‐PAX2 and the envelope plasmid pMD2G into HEK‐293FT cells using Lipofectamine 3000 (Life Technologies, Carlsbad, CA, USA) according to the manufacturer's instructions. AGS and HGC‐27 cells were infected with recombinant lentivirus‐transducing units (pHBLV‐PTTG3P or empty vector) plus 6 μg/ml polybrene (Sigma‐Aldrich, St. Louis, MO, USA). After incubation for 48–72 hrs, the cells were harvested for RNA, and protein extraction or were resuspended for other assays. Stably infected cells were maintained with 2 μg/ml puromycin (Sigma‐Aldrich).

### Cell proliferation assays

Cell proliferation was evaluated using Cell Counting Kit‐8 (CCK‐8) (Dojindo, Kumamoto, Japan), an EdU DNA imaging kit (Life Technology) and colony‐formation assays. The former two assays were performed on 2 × 10^3^ cells grown in 96‐well plates according to the recommended protocols. An automatic microplate reader (BioTek, Winooski, VT, USA) was used to determine the absorbance at 450 nm. Images of the cells used in the EdU assays were taken at 100× and counted at 200× under an immunofluorescence microscope (Olympus, Tokyo, Japan).

For colony‐formation assays, 800 cells were seeded onto 6‐well plates and incubated for 2 weeks. Then, the cells were fixed with ethanol and stained with crystal violet. The number of colonies containing more than 30 cells was counted.

### Analysis of apoptosis and cell cycle

Flow cytometry assays were performed to analyse cell cycle progression and apoptosis. After cells were incubated for 48 hrs, they were stained with Annexin V‐FITC and/or propidium iodide (PI) (BD Bioscience, USA) for apoptosis analysis or fixed with ethanol overnight at −20°C followed by subsequent PI (Calbiochem) staining for DNA content (cell cycle) analysis.

### Cell motility and invasion assays

A wound‐healing assay was performed to assess cell motility. Transfected cells were plated at equal densities in 6‐well plates and grown to 100% confluence. The cells were pre‐treated with Mitomycin C (Sigma‐Aldrich) for 1 hr at 37°C to separate the role of cell motility from that of cell proliferation. Wounds were scratched with sterile pipette tips, loose cells were removed by rinsing with PBS, and serum‐free medium was added. The wounds were observed at 0 and 6 hrs after scratching under a microscope at 100× (Olympus).

Transwell chambers (8 μm, 24‐well format) (Corning) were employed for cell invasion assays. A total of 4 × 10^4^ cells in 100 μl of serum‐free medium was loaded into the upper inserts, and 500 μl of culture medium containing 10% FBS was loaded into the lower chambers as a chemo‐attractant. After a 24‐hr incubation at 37°C, the cells that had migrated through the filters were fixed with ethanol and stained with crystal violet. Photographs were taken under a microscope at 200× (Olympus), and the number of invaded cells was counted at 400×.

### Tumour formation and metastasis assays in a nude mouse model

Athymic female BALB/c nude mice were maintained under specific‐pathogen‐free conditions. HGC‐27 cells stably expressing PTTG3P or the vector control were harvested and resuspended with RMPI‐1640. For tumour formation, a total of 5 × 10^6^ cells were subcutaneously injected into the right flank of each 5‐week‐old mouse (five mice for each group). For tumour metastasis, 1 × 10^6^ cells were injected into the tail vein of each mouse (three mice per group). After transplantation, the weight of the mice and the growth of the subcutaneous tumours were assessed every 2 days. The mice were killed after a period of 4–6 weeks. Subcutaneous tumours and lungs were excised and measured. Tumour size was measured with the following formula: (*L* × *W* ^2^)/2, where *L* is the length and *W* is the width of each tumour.

### Western blotting

Cells were lysed in RIPA buffer (Sigma‐Aldrich) supplemented with a protease inhibitor (Roche, Basel, Switzerland) and a phosphatase inhibitor (Roche). Protein concentration was measured using a BCA protein assay kit (Thermo Scientific, USA). Antibodies against PARP1 (#9542), cleaved PARP1 (#5625), caspase‐3 (#9665), cleaved caspase‐3 (#9664), cyclin D1 (#2978), p27 (#2552) and GAPDH (#2118) were purchased from Cell Signaling Technology (Cambridge, MA, USA). Isolated proteins were probed with the indicated primary antibodies followed by incubation with HRP‐linked secondary antibodies and detection using an ECL system (Thermo Fisher, USA). Protein expression levels were normalized to that of GAPDH (Cell Signaling Technology).

### Statistical analysis

All statistical analyses were performed using SPSS 20.0 (IBM, Chicago, IL, USA).

Correlations between PTTG3P expression and clinicopathological parameters were analysed using the Chi‐square test. PTTG3P expression was assessed using the Chi‐square test or Fisher's exact probability test. Survival was calculated using the Kaplan–Meier method and compared with the log‐rank test. The results of the functional assays were analysed using Student's *t*‐test. Variables with a value of *P* < 0.05 in univariate analysis were used in multivariate analysis based on the Cox proportional hazards model. *P* values less than 0.05 were considered significant.

## Results

### PTTG3P is up‐regulated in GC tissues and correlates with poor prognosis

**Figure 1:**
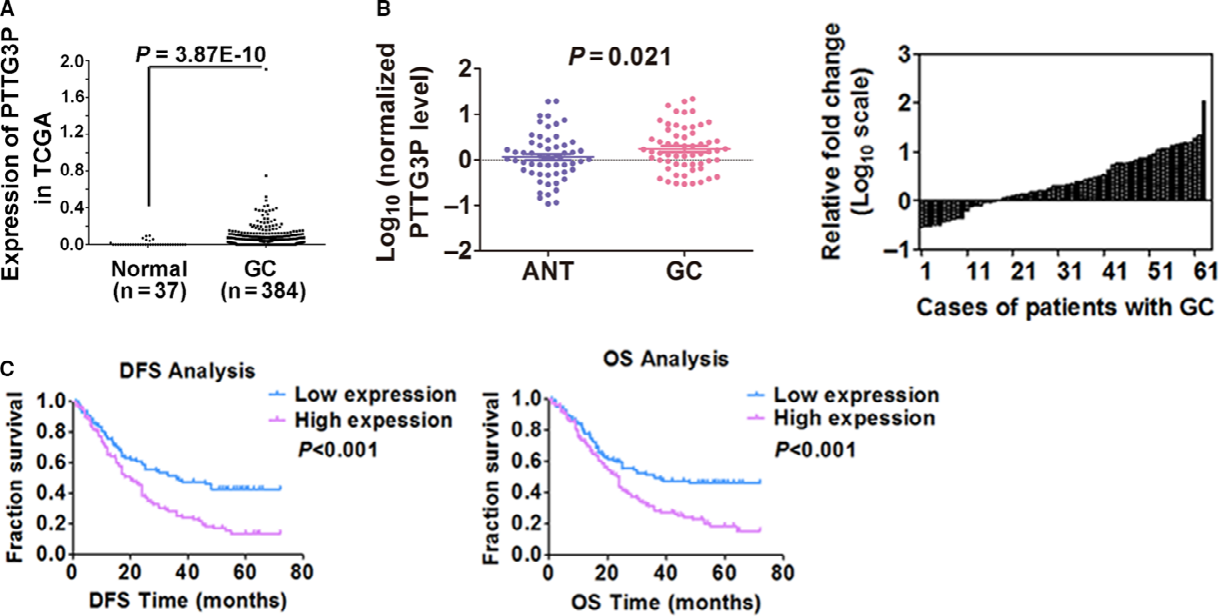
PTTG3P is up‐regulated in GC tissues and is correlated with patient prognosis. (**A**) PTTG3P expression was evaluated using TCGA RNA‐seq data and compared between GC tissues and normal tissues. (**B**) The expression of PTTG3P in adjacent non‐tumour (ANT) and GC tissues was determined by qRT–PCR. β‐actin was used as an endogenous control to normalize the data. (**C**) Kaplan–Meier curves for DFS and OS of patients with GC based on PTTG3P expression.

We previously identified systemic variations in lncRNA expression between GC and paired non‐tumour samples performed with microarray analysis 12 and noted that the pseudogene PTTG3P was up‐regulated (2.008‐fold change; *P* = 0.022) in GC tissues. A similar result was also found in *The Cancer Genome Atlas* (TCGA) database (*P* = 3.87E−10, Fig. 1A). Therefore, we analysed the mRNA expression levels of PTTG3P in 63 pairs of GC tissues and adjacent non‐tumours (ANTs) and found that PTTG3P was significantly up‐regulated in 68.3% (43 of 63) of the GC tissues compared with the ANTs (*P* = 0.021, Fig. 1B). We next analysed the correlation between PTTG3P expression and clinicopathological characteristics in another 136 patients with GC. As shown in Table 1, high PTTG3P expression levels divided by the median value 14 were tightly correlated with larger tumour sizes (*P* = 0.043) and higher recurrence rates (*P* = 0.022).

**Table 1:** Relationship between PTTG3P expression and histopathological factors in patients with gastric cancer

Characteristics	Number (*n* = 198)	%	PTTG3P expression		*P*
			Low	High	
Age (years)					0.152
<60	86	43.43	38	48	
≥60	112	56.57	61	51	
Gender					0.192
Male	148	74.75	78	70	
Female	50	25.25	21	29	
Size (cm)					0.043^a^
<5	116	58.59	65	51	
≥5	82	41.41	34	48	
Differentiation					0.185
Poor and other	150	75.76	71	79	
Well and moderate	48	24.24	28	20	
Lymphatic invasion					0.107
Absent	75	37.88	43	32	
Present	123	62.12	56	67	
Nerve invasion					0.300
Absent	71	35.86	39	32	
Present	127	64.14	60	67	
T grade					0.294
T1	3	1.52	3	0	
T2	16	8.08	7	9	
T3	25	12.63	14	11	
T4	154	77.78	75	79	
LNM					0.103
Absent	28	14.14	18	10	
Present	170	85.86	81	89	
TNM					0.468
I	10	5.05	6	4	
II	31	15.66	19	12	
III	137	69.19	65	72	
IV	20	10.10	9	11	
Recurrence					0.022^a^
Absent	110	55.56	63	47	
Present	88	44.44	36	52	

LNM: lymph node metastasis.

a *P* < 0.05.

DFS and OS curves were plotted according to PTTG3P expression level by the Kaplan–Meier method and log‐rank tests. As shown in Figure 1C, high expression of PTTG3P was correlated with both significantly shorter DFS (*P* < 0.001) and OS (*P* < 0.001; Fig. 1C). Univariate analysis of survival revealed that the relative level of PTTG3P expression (*P* < 0.001), tumour grade (*P* < 0.001), lymphatic metastasis (*P* = 0.002) and TNM stage (*P* < 0.001) was prognostic indicators of DFS (Table 2) and OS (Table 3). Moreover, multivariate Cox regression analysis showed that both advanced TNM stage and high expression of PTTG3P were independent risk factors of DFS (Table 2) and OS (Table 3; *P* < 0.05). These results suggest that PTTG3P is a potential independent prognostic factor for GC.

**Table 2:** Univariate and multivariate analyses of clinicopathological factors associated with disease‐free survival in gastric cancer patients

Characteristics	Univariate analysis		Multivariate analysis	
	HR (95% CI)	*P*	HR (95% CI)	*P*
Tumour grade (T1, T2, T3, T4)	1.907 (1.366–2.663)	0.000^a^		
LNM (present/absent)	2.541 (1.403–4.602)	0.002^a^		
TNM (I, II, III, IV)	2.015 (1.508–2.694)	0.000^a^	1.553 (1.058–2.279)	0.025^a^
PTTG3P (high/low)	1.887 (1.343–2.653)	0.000^a^	1.684 (1.195–2.374)	0.003^a^

HR: hazard ratio; CI: confidence interval; LNM: lymph node metastasis.

a *P* < 0.05.

**Table 3:** Univariate and multivariate analyses of clinicopathological factors associated with overall survival in gastric cancer patients

Characteristics	Univariate analysis		Multivariate analysis	
	HR (95% CI)	*P*	HR (95% CI)	*P*
Tumour grade (T1, T2, T3, T4)	3.025 (1.333–6.868)	0.008^a^	–	–
LNM (present/absent)	3.132 (1.590–6.171)	0.001^a^	–	–
TNM (I, II, III, IV)	2.079 (1.549–2.792)	0.000^a^	1.619 (1.104–2.373)	0.014^a^
PTTG3P (high/low)	1.794 (1.266–2.541)	0.001^a^	1.578 (1.110–2.243)	0.011^a^

HR: hazard ratio; CI: confidence interval; LNM: lymph node metastasis.

a *P* < 0.05.

### PTTG3P stimulates GC tumour cell proliferation

**Figure 2:**
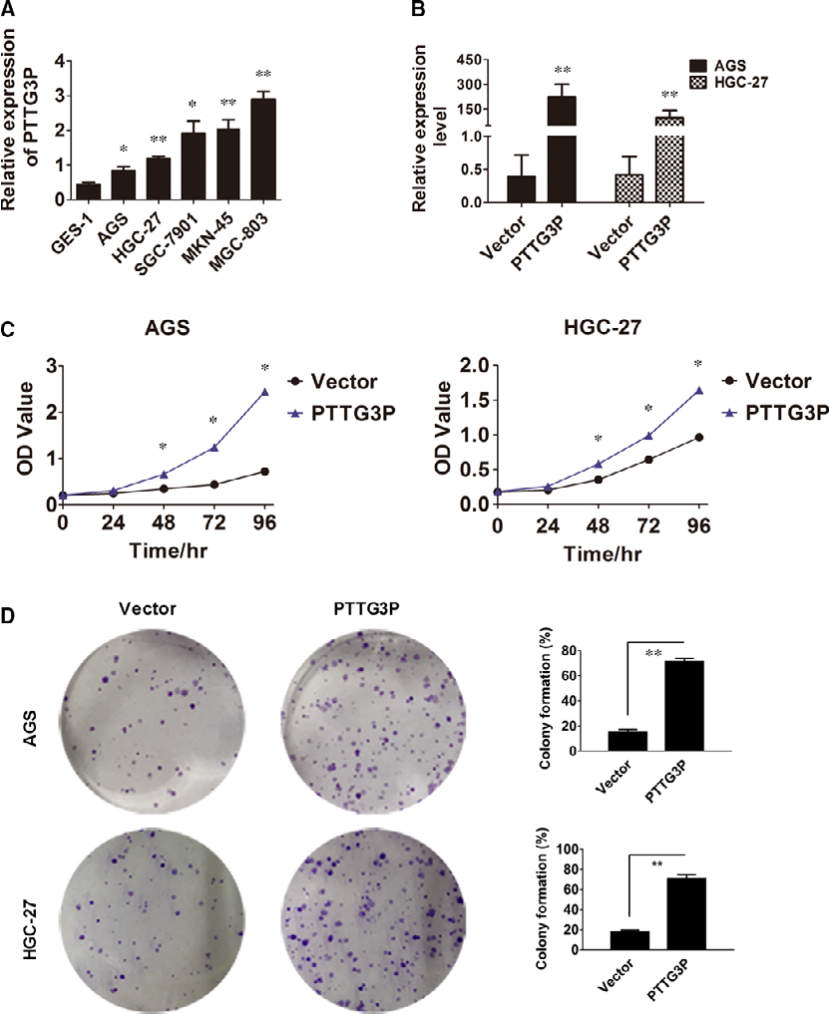
PTTG3P stimulates GC tumour cell proliferation. (**A**) PTTG3P expression was quantitated in 5 GC cell lines and the GES‐1 cell line using qRT–PCR. (**B**) The GC cell lines AGC and HGC‐27 were transfected with either PTTG3P or a vector control, and PTTG3P overexpression was verified by qRT–PCR. (**C**) Cell viability of AGS and HGC‐27 cells transfected with PTTG3P or a vector control was measured using a CCK8 assay. (**D**) The cell colony‐formation ability of AGS and HGC‐27 cells transfected with PTTG3P or the vector control was measured. Data are shown as the mean ± SD of three replicates; *P < 0.05 and **P < 0.01.

To investigate the biological effects of PTTG3P in GC progression, baseline levels of PTTG3P expression were examined in five GC cell lines and a normal human gastric epithelial cell line. PTTG3P expression was significantly elevated in the GC cell lines (Fig. 2A). AGS and HGC‐27 cells were selected for overexpression experiments, and the efficiency of overexpression was validated by qRT–PCR (Fig. 2B). Then, we utilized CCK8 and colony‐formation assays to elucidate the potential effect of PTTG3P on GC tumour cell proliferation. The CCK8 assay showed that the absorbance of PTTG3P‐overexpressing cells was significantly higher than that of vector control‐transfected GC cells, suggesting that overexpression of PTTG3P in AGS and HGC‐27 cells leads to increased accumulation of living tumour cells (*P* < 0.05; Fig. 2C). Similarly, the colony‐formation assays showed that overexpression of PTTG3P led to the formation of more colonies in both AGS and HGC‐27 cells than in controls (*P* < 0.01; Fig. 2D). These data suggest that PTTG3P stimulates GC tumour cell proliferation.

### PTTG3P promotes G_1_–S cell cycle transition in GC cells

**Figure 3:**
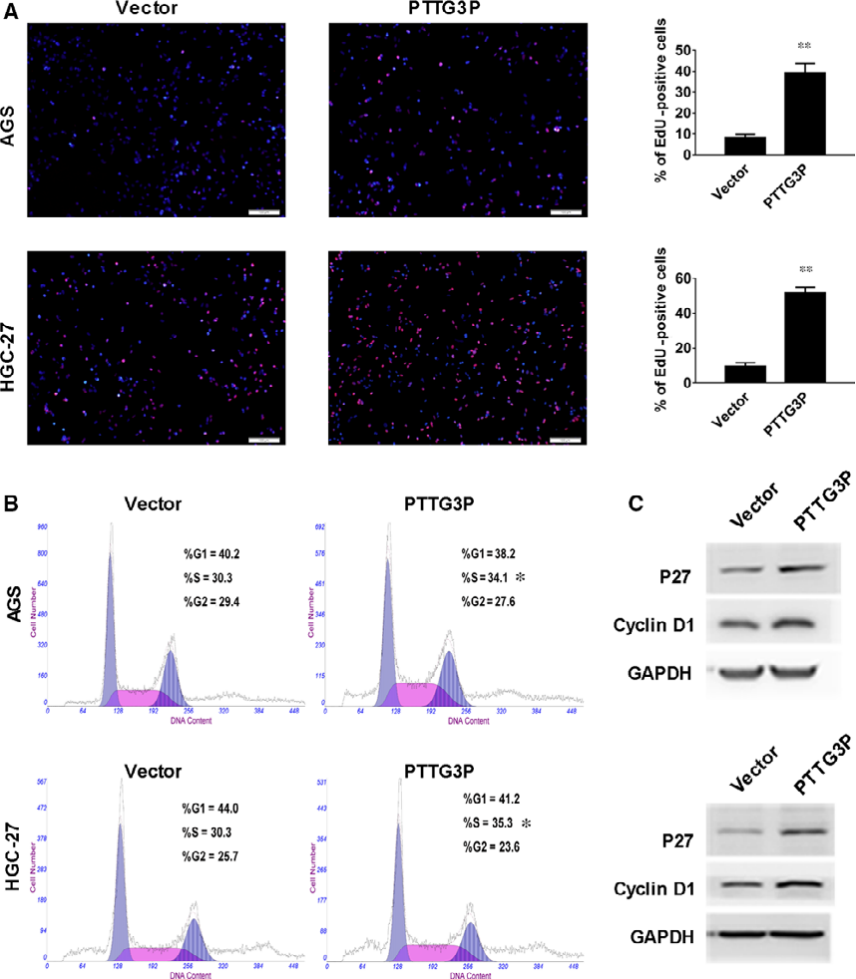
PTTG3P promotes G1‐S cell cycle transition in GC cells. (**A**) EdU incorporation was evaluated using an EdU imaging kit. Quantitative analysis showed a greater number of EdU‐positive cells in the PTTG3P‐overexpression group compared to the control. Cells were counted by immunofluorescence microscopy under 200× magnification (scale bars = 100 μm). (**B**) Cell cycle distribution of AGS and HGC‐27 cells transfected with PTTG3P or a vector control was evaluated using a flow cytometer. (**C**) Western blotting analysis of the expression levels of cyclin D1 and p27. GAPDH was used as a reference. Data are shown as the mean ± SD of three replicates; **P < 0.01.

Next, we performed EdU immunofluorescence and flow cytometry assays to further explore the potential influence of PTTG3P on cell cycle progression in GC cells. An EdU labelling assay showed a greater number of EdU‐positive cells in cultures overexpressing PTTG3P than in the vector group, suggesting that the up‐regulation of PTTG3P caused more cells to enter S phase compared to the control (Fig. 3A). Furthermore, cell cycle distribution analysis also showed that the percentage of PTTG3P‐overexpressing AGS and HGC‐27 cells in S phase (34.1% and 35.3%, respectively) was significantly higher than that in the control group (30.3% and 30.3%, respectively; *P* < 0.05), coupled with a decrease in the number of cells in G_1_ phase. These results suggest that PTTG3P might affect the G_1_–S transition (Fig. 3B). Finally, Western blotting results also demonstrated that overexpression of PTTG3P increased cyclin D1 and p27 protein levels in AGS and HGC‐27 cells (Fig. 3C). Overall, these data suggest that PTTG3P prompts cell cycle progression by promoting the G_1_–S transition in GC cells.

### PTTG3P inhibits GC tumour cell apoptosis

**Figure 4:**
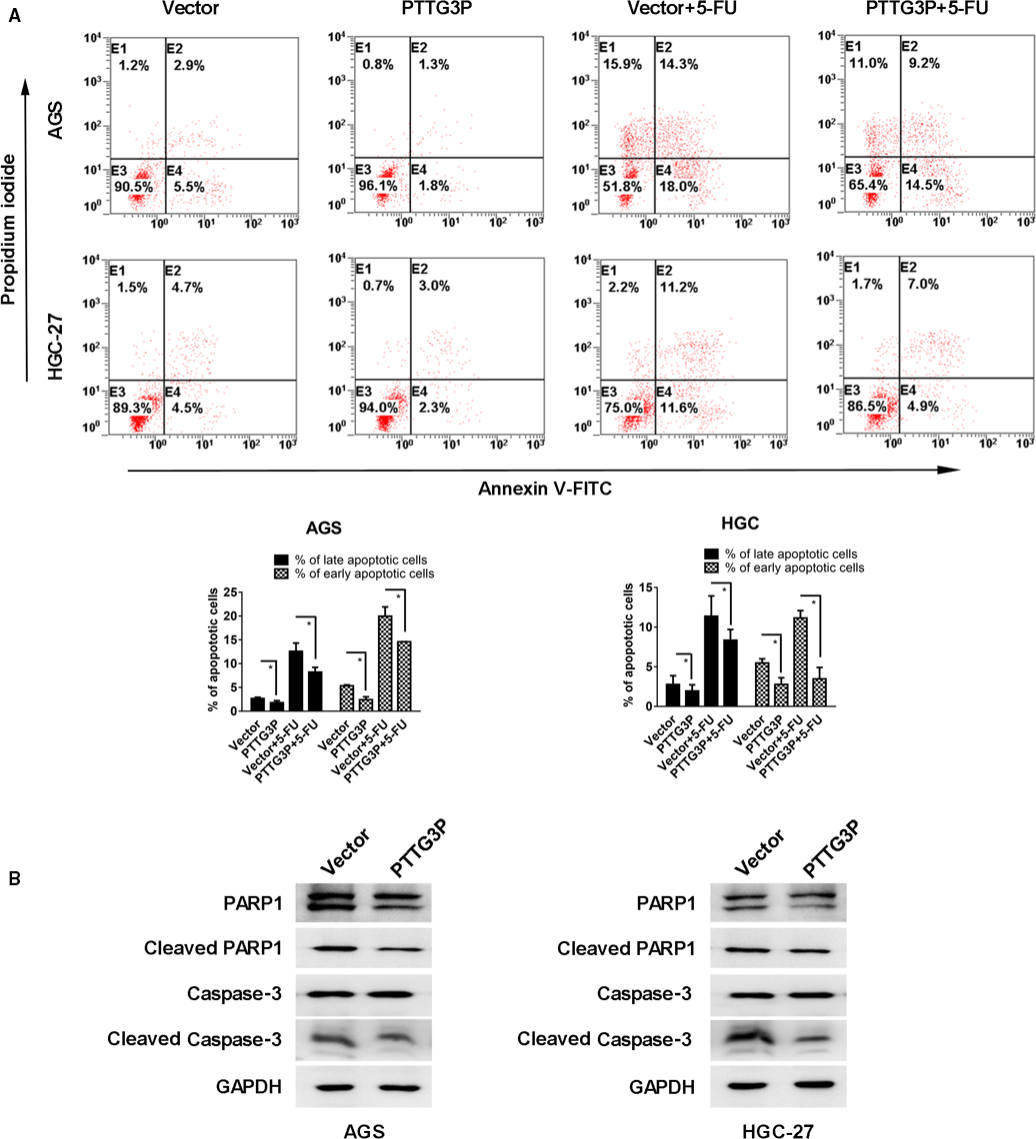
PTTG3P inhibits GC tumour cell apoptosis. (**A**) GC cells were transfected with PTTG3P or control vector and treated with or without 5‐FU for 24 hrs. Then, all the cells were stained with Annexin V and PI. The Annexin V^−^PI^−^, Annexin V^+^PI^−^, Annexin V^−^PI^+^ and Annexin V^+^PI^+^ subgroups indicate live cells, early apoptotic cells, necrotic cells and late apoptotic cells, respectively. (**B**) Western blotting analysis of the expression levels of PARP1, cleaved PARP1, caspase‐3 and cleaved caspase‐3. GAPDH was used as a reference. Data are shown as the mean ± SD of three replicates; *P < 0.05.

We also performed an Annexin V apoptosis assay to determine the influence of PTTG3P on GC cell apoptosis. The results showed that after overexpression of PTTG3P, both AGS and HGC‐27 cells showed significantly decreased proportions of early apoptotic cells (Annexin V^+^PI^−^) (1.8% and 2.3%, respectively) and late apoptotic cells (Annexin V^+^PI^+^) (1.3% and 3.0%, respectively) compared with controls (early apoptotic cells, 5.5% and 4.5%; late apoptotic cells, 2.9% and 4.7%, respectively; *P* < 0.05; Fig. 4A). Next, a 5‐FU‐induced apoptosis assay was performed to confirm the influence of PTTG3P on cell apoptosis, and similar results were obtained. Western blotting showed that overexpression of PTTG3P attenuated PARP1 and caspase‐3 cleavage in both AGS and HGC‐27 cells (Fig. 4B). Thus, PTTG3P reduces GC tumour cell apoptosis.

### PTTG3P overexpression promotes GC cell migration and invasion

**Figure 5:**
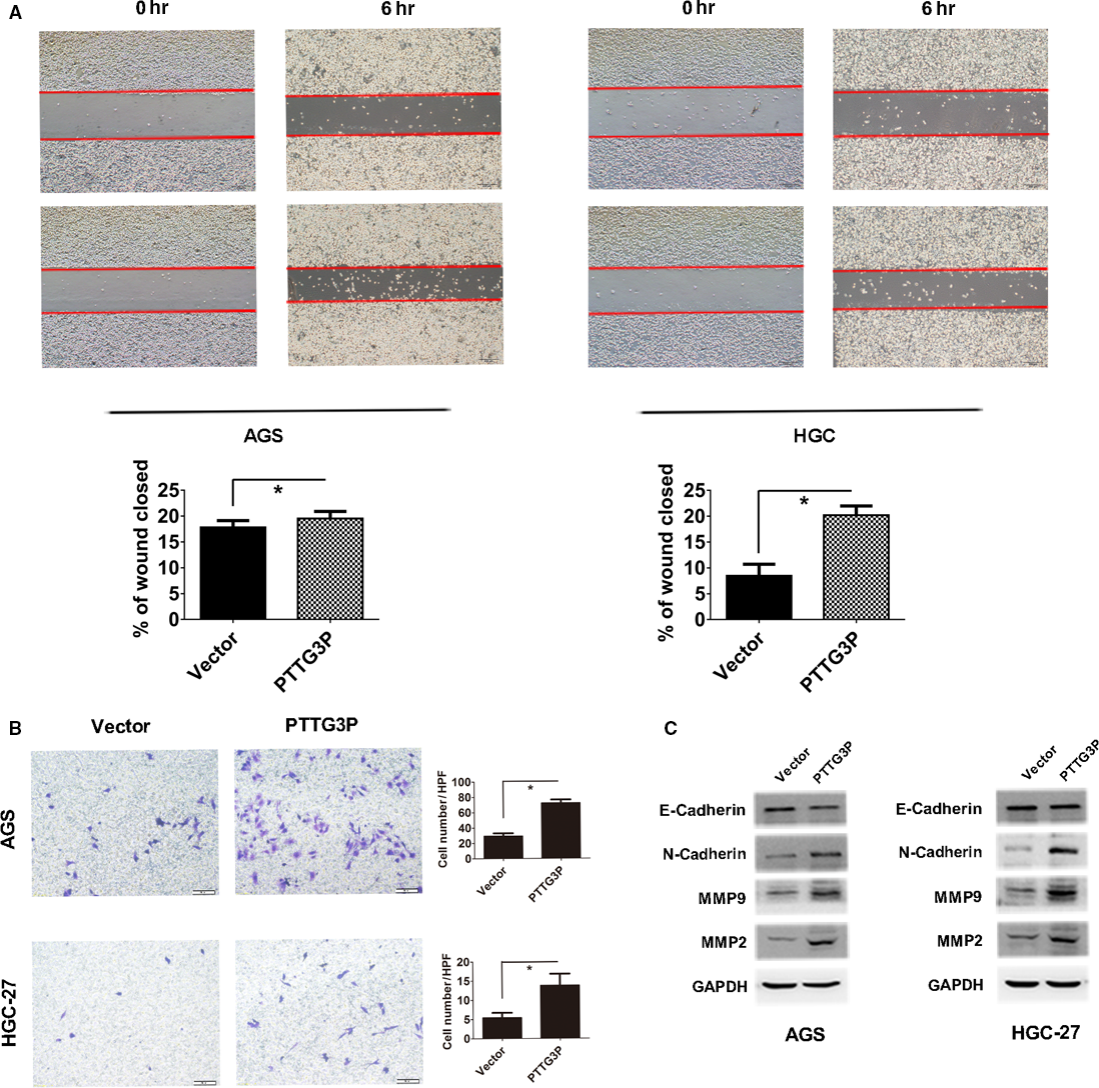
PTTG3P promotes GC cell migration and invasion. (**A**) Cell migration ability was evaluated using a wound‐healing assay; images of AGS and HGC‐27 cells were taken at 0 and 6 hrs post‐scratch. (**B**) Cell invasion potential in AGS and HGC‐27 cells was assessed using a transwell assay. (**C**) Western blotting analysis of the expression levels of E‐cadherin, N‐cadherin, MMP2 and MMP9. GAPDH was used as a reference. Data are shown as the mean ± SD of three replicates; *P < 0.05.

To determine whether PTTG3P promotes GC cell invasion and migration, we performed wound healing and transwell assays. Overexpression of PTTG3P resulted in accelerated wound‐healing rates (*P* < 0.05; Fig. 5A) and an increased number of invaded cells (*P* < 0.01; Fig. 5B) in both AGS and HGC‐27 cells. Accordingly, the Western blotting results showed that overexpression of PTTG3P up‐regulated invasion‐promoting proteins such as N‐cadherin, MMP2 and MMP9 and down‐regulated the invasion‐inhibiting protein E‐cadherin (Fig. 5C). Collectively, these results suggest that PTTG3P enhances GC cell migration and invasion.

### PTTG3P promotes GC tumour growth and metastasis *in vivo*

**Figure 6:**
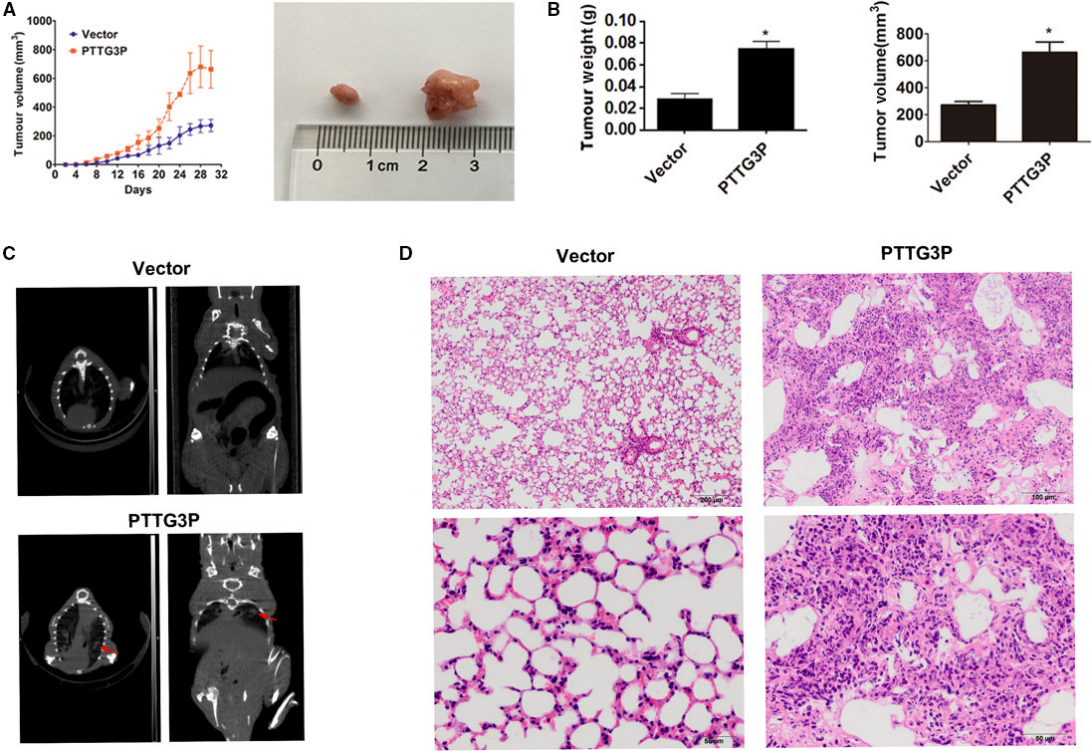
PTTG3P promotes GC tumour growth and metastasis *in vivo*. (**A**) Tumour volumes of xenograft models were measured every 4 days. Representative photographs of tumour macroscopic appearance are shown. (**B**) Tumour size and body weight in the PTTG3P‐overexpression xenograft group compared with the control. (**C**) CT scanning of the metastasis models. Red arrows denote potential pulmonary metastases in PTTG3P‐overexpressing nude mice. (**D**) H&E staining was used to verify the formation of pulmonary metastatic tumours in the PTTG3P‐overexpression group (top scale bars = 200 μm; bottom scale bars = 50 μm). Data are shown as the mean ± SD of three replicates; *P < 0.05.

Finally, we used nude mouse xenograft and metastasis models to investigate the functions of PTTG3P *in vivo*. Consistent with the *ex vivo* data, overexpression of PTTG3P remarkably accelerated tumour growth compared with controls in the xenograft models (Fig. 6A). In addition, the xenografts derived from PTTG3P‐overexpressing HGC‐27 cells exceeded those derived from control cells in both size and weight (Fig. 6B). Metastasis assays showed that potential pulmonary metastases could be detected by CT scanning in the PTTG3P overexpression group (Fig. 6C bottom, red arrows indicate the suspicious tumour masses), whereas tumour masses were not detected in the empty‐vector control group (Fig. 6C top), which was further confirmed by H&E staining (Fig. 6D). These results suggest that PTTG3P facilitates GC tumour growth and metastasis *in vivo*.

### PTTG3P expression is independent of its parent genes PTTG1 and PTTG2

**Figure 7:**
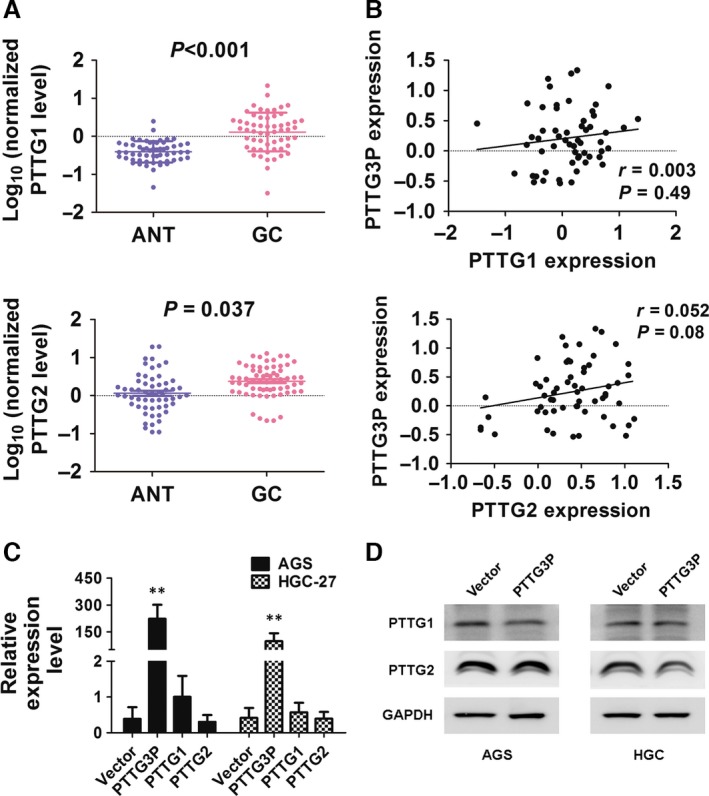
PTTG3P overexpression is not correlated with PTTG1 or PTTG2 expression. (**A**) The expression of PTTG1 and PTTG2 in adjacent non‐tumour (ANT) and GC tissues was determined by qRT–PCR. (**B**) Correlations between PTTG3P expression and PTTG1 or PTTG2 expression were evaluated. (**C**) mRNA expression levels of PTTG1 and PTTG2 in cells overexpressing PTTG3P were evaluated by qRT–PCR. (**D**) Protein expression levels of PTTG1 and PTTG2 in cells overexpressing PTTG3P were evaluated by Western blotting; GAPDH was used as a reference. Data are shown as the mean ± SD of three replicates; **P < 0.01.

Because the parent genes PTTG1 and PTTG2 were previously confirmed to be oncogenes in various cancer types 11, 15, 16, 17 and PTTG3P shares high similarity with these genes (Fig. S1), we suggested that PTTG3P plays a role in modulating PTTG1 or PTTG2 that is similar to previously reported mechanisms 18, 19. To test this hypothesis, we designed specific primer sets for PTTG3P, PTTG1 and PTTG2. PCR products were examined by agarose gel electrophoresis followed by sequencing to confirm primer specificity (Fig. S2). Similar to PTTG3P, both PTTG1 and PTTG2 were up‐regulated in GC tissues compared with ANTs (Fig. 7A), but correlation analysis showed that the expression levels of these two parent genes were not related to PTTG3P expression (*P* > 0.05, Fig. 7B). *In vitro* overexpression of PTTG3P in GC cell lines also failed to significantly alter the mRNA or protein expression level of either PTTG1 or PTTG2 (Fig. 7C and D). Thus, we concluded that PTTG3P expression and function are independent of its true gene counterparts.

## Discussion

To the best of our knowledge, our study is the first to clarify the biological functions of PTTG3P in somatic malignancies, and to demonstrate that its oncogenic functions occur independently of its parent genes. We found that PTTG3P expression was elevated in GC tissues at the mRNA level and that PTTG3P served as an independent prognostic factor for GC. Up‐regulation of PTTG3P promoted *in vitro* and *in vivo* GC cell proliferation and invasion/metastasis. Based on these results, we identified PTTG3P as a functional oncogenic pseudogene and a possible negative prognostic predictor of GC.

It was previously reported that PTTG3P is expressed at extremely low levels or is absent in normal tissues but is detectable in ovarian tumour tissues and cell lines 8. In the present study, we showed that PTTG3P was significantly overexpressed in GC tissues compared with ANTs, suggesting that this pseudogene has a cancer‐specific expression pattern in GC tissues. Clinically, the up‐regulation of PTTG3P was highly correlated with increased tumour burden and a higher after treatment recurrence rate, suggesting that PTTG3P facilitates tumour progression by enhancing tumour cell proliferation and invasion. This hypothesis was verified in subsequent *in vitro* cell experiments and nude mouse models. The enhanced tumour cell proliferation and invasiveness caused by abnormally high expression of PTTG3P thus resulted in more aggressive biological behaviour, leading to shortened DFS and OS in patients with GC. Future studies might verify the clinical significance of PTTG3P expression in a larger number of GC samples to identify its prognostic value for patients with GC.

As a member of the PTTG family, PTTG3P is recognized as an intronless pseudogene that shares high homology with its parent genes PTTG1 and PTTG2, which led us to presume that PTTG3P might exert its biological effect *via* PTTG1/2 modulation, similar to other reported pseudogenes 18, 19. However, correlation analysis of PTTG3P with PTTG1 and PTTG2 expression in GC tissue did not reveal a significant relationship. Additionally, *in vitro* overexpression of PTTG3P in GC cell lines failed to alter the expression of the other two genes at the mRNA and protein levels. These results suggest that PTTG3P exerts its oncogenic role independently of its parent genes.

Because pseudogenes might not possess transcriptional control regions, they may be subject to transcriptional elements that are different from paralogous functional genes, thus exerting different functions than the parent genes 20, 21. Poliseno and colleagues revealed that the PTENP1 3′UTR significantly suppressed cell proliferation in PTEN‐null PC3 cells, supporting the notion that PTENP1 could exert a tumour‐suppressive role independent of the parent PTEN gene 2. Likewise, ψPPM1K, a partial retrotranscript pseudogene containing inverted repeats capable of being processed into two endo‐siRNAs, regulates cell growth‐related target genes and exerts tumour‐suppressive activity independent of its cognate gene PPM1K 3. The pseudogene ψCx43 can be translated into a 43 kD protein that bears an amino acid change from arginine R202 to cysteine, resulting in a deficiency in the ability to mediate intercellular communication 22. Collectively, these results suggest that pseudogenes, especially those subject to different transcriptional elements, may exert their biological roles independently of their parent genes. Furthermore, PTTG3P is located at 8q13.1, whereas the ancestral genes are located on chromosomes 5 and 4 8; therefore, it is possible that PTTG3P might be subject to different transcriptional regulation and might possess a different regulatory function compared to those of its cognate counterpart, thus allowing it to exert its oncogenic role independently of its parent genes.

In summary, the current study suggests that PTTG3P exhibits a novel oncogenic role in the regulation of pathways related to cell cycle progression that is independent of its parent gene and that this pseudogene predicts poor prognosis in patients with GC. Our findings provide new insights into the molecular details of GC and the potential therapeutic targets that can be used to combat this disease.

## Conflict of interest

The authors declare that they have no conflicts of interest.

## Supporting information


**Figure S1** The homologous sequences of PTTG3P, PTTG1, and PTTG2. Black represents the matched nucleotides among PTTG3P, PTTG1 and PTTG2, while white represents the differences

**Figure S2** Verifying the qRT‐PCR primers. (A) After designing specific primer sets for PTTG3P, PTTG1, and PTTG2, PCR was performed to verify the efficacy of the primers. (B) Sequencing the PCR product to verify the specificity of PTTG3P primers. (C) Sequencing the PCR product to verify the specificity of PTTG1 primers. (D) Sequencing the PCR product to verify the specificity of PTTG2 primers
