# Supplementary figures and images for: PTTG3P promotes gastric tumour cell proliferation and invasion and is an indicator of poor prognosis
Source: J Cell Mol Med. 2017 Jun 19;21(12):3360–71. doi: 10.1111/jcmm.13239 (PMC5706523; doi:10.1111/jcmm.13239)

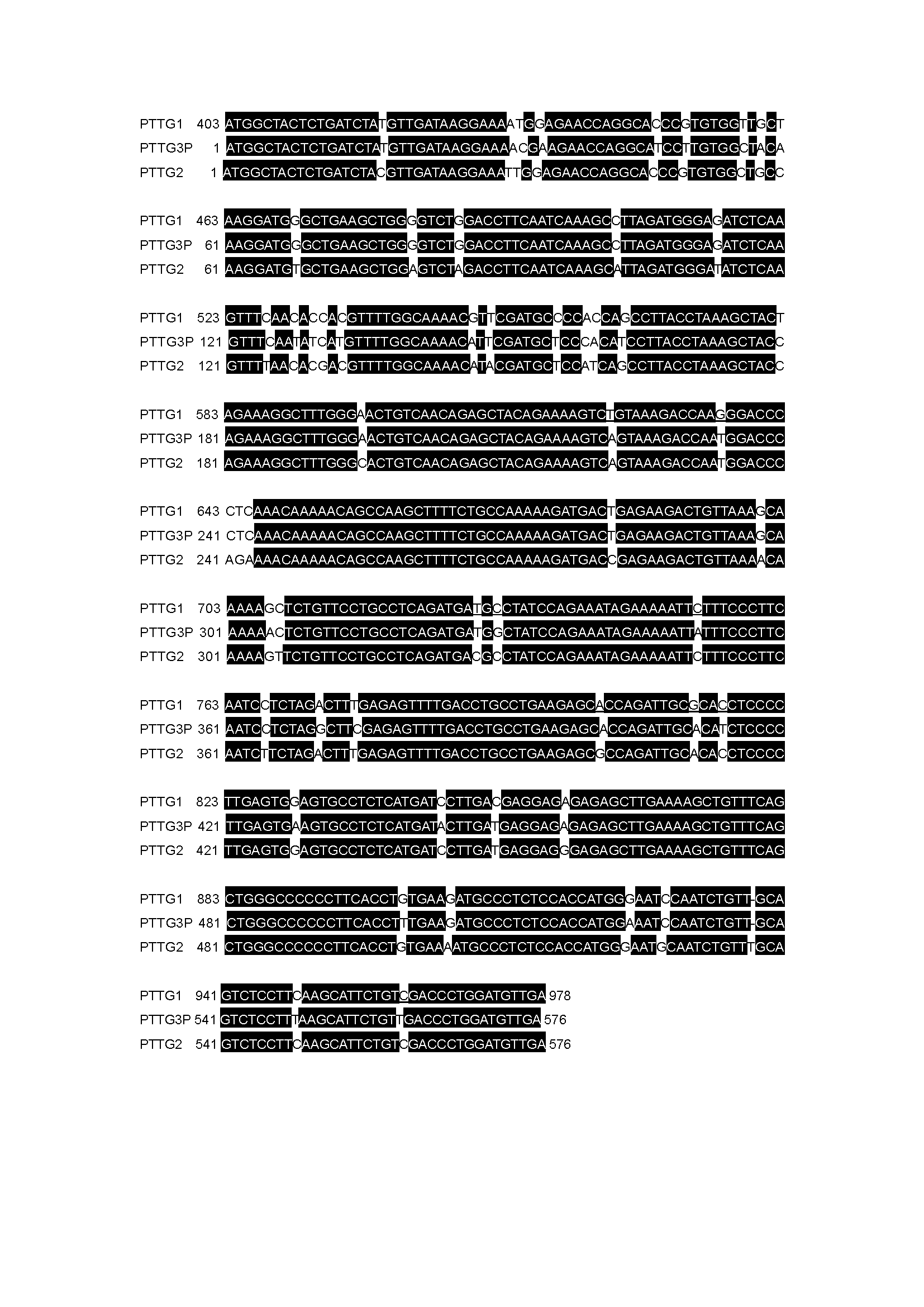

Supplement: Supplementary file 1 — Figure S1 The homologous sequences of PTTG3P, PTTG1, and PTTG2. Black represents the matched nucleotides among PTTG3P, PTTG1 and PTTG2, while white represents the differences [file JCMM-21-3360-s001.tif]

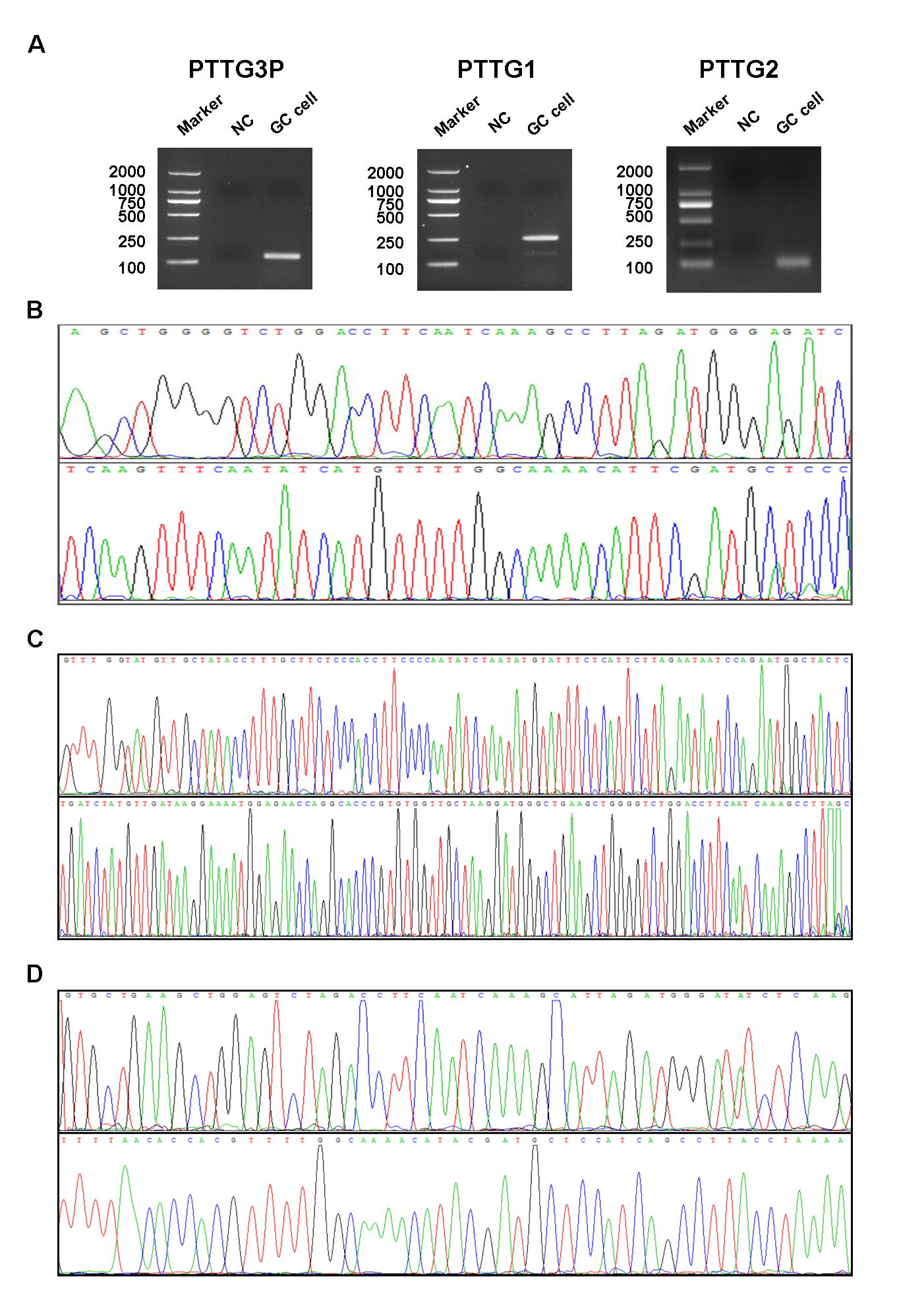

Supplement: Supplementary file 2 — Figure S2 Verifying the qRT‐PCR primers. (A) After designing specific primer sets for PTTG3P, PTTG1, and PTTG2, PCR was performed to verify the efficacy of the primers. (B) Sequencing the PCR product to verify the specificity of PTTG3P primers. (C) Sequencing the PCR product to verify the specificity of PTTG1 primers. (D) Sequencing the PCR product to verify the specificity of PTTG2 primers [file JCMM-21-3360-s002.tif]
